# Breast cancer mortality in participants of the Norwegian Breast Cancer Screening Program

**DOI:** 10.1002/cncr.28174

**Published:** 2013-05-29

**Authors:** Solveig Hofvind, Giske Ursin, Steinar Tretli, Sofie Sebuødegård, Bjørn Møller

**Affiliations:** 1 Department of Research, Cancer Registry of NorwayOslo, Norway; 2 Oslo and Akershus University College of Applied SciencesOslo, Norway; 3 Cancer Registry of NorwayOslo, Norway; 4 Institute of Basic Medical Sciences, University of OsloOslo, Norway; 5 Department of Preventive Medicine, University of Southern CaliforniaLos Angeles, California; 6 The Norwegian University of Science and TechnologyTrondhjem, Norway; 7 Department of Registration, Cancer Registry of NorwayOslo, Norway

**Keywords:** mammography, mass screening, female, breast neoplasms/mortality, breast neoplasms/prevention and control, breast neoplasms/radiography, Norway/epidemiology

## Abstract

**BACKGROUND:**

The Norwegian Breast Cancer Screening Program started in 1996. To the authors' knowledge, this is the first report using individual-based data on invitation and participation to analyze breast cancer mortality among screened and nonscreened women in the program.

**Methods:**

Information on dates of invitation, attendance, breast cancer diagnosis, emigration, death, and cause of death was linked by using unique 11-digit personal identification numbers assigned all inhabitants of Norway at birth or immigration. In total, 699,628 women ages 50 to 69 years without prior a diagnosis of breast cancer were invited to the program from 1996 to 2009 and were followed for breast cancer through 2009 and death through 2010. Incidence-based breast cancer mortality rate ratios (MRRs) were compared between the screened and nonscreened cohorts using a Poisson regression model. The MRRs were adjusted for calendar period, attained age, years since inclusion in the cohorts, and self-selection bias.

**RESULTS:**

The crude breast cancer mortality rate was 20.7 per 100,000 women-years for the screened cohort compared with 39.7 per 100,000 women-years for the nonscreened cohort, resulting in an MRR of 0.52 (95% confidence interval, 0.47-0.59). The mortality reduction associated with attendance in the program was 43% (MRR, 0.57; 95% confidence interval, 0.51-0.64) after adjusting for calendar period, attained age, years after inclusion in the cohort, and self-selection bias.

**CONCLUSIONS:**

After 15 years of follow-up, a 43% reduction in mortality was observed among women who attended the national mammographic screening program in Norway.

## INTRODUCTION

The objective of mammographic screening is to detect breast cancer at an early stage and thereby reduce mortality from the disease. A beneficial effect from such screening was observed in several randomized controlled trials^1^–^3^ and most recently in the analyzes of the European service screening programs^4^,^5^ and in a review by an independent panel in the United Kingdom of the benefits and harms of breast cancer screening.^3^

The extent of mortality reduction after the implementation of organized service screening has been debated for decades.^1^,^2^,^6^ Some issues that have been discussed are the study design, the estimation methods, the required length of follow-up, and the effects of changes in treatment over time.^7^–^12^

The Euroscreen Working Group estimated a 25% reduction in breast cancer mortality in cohort studies and a 31% reduction in case-control studies among women who were invited versus noninvited to service screening programs,^4^ whereas the UK independent panel reported a reduction in breast cancer mortality of 20%, based on randomized controlled trials.^3^ Two previous studies used individual cancer data but aggregated screening data to address the effect of the Norwegian Breast Cancer Screening Program (NBCSP) among women who were invited to the program.^9^,^13^ Those studies reported 10% and 11% reductions in breast cancer mortality associated with being invited to screening. The use of aggregated screening data and short follow-up may explain the low estimates in the 2 studies.

To take advantage of the individual level data on invitation and screening history, we used a cohort study design and an incidence-based approach to analyze breast cancer mortality among women who were invited to the screening program in Norway. This is the first mortality analysis from the program using these individual level screening data. We specifically tested the effect of attending the program on breast cancer mortality after adjusting for calendar period, attained age, time since inclusion in the cohort, and self-selection bias.

## MATERIALS AND METHODS

### Methods

**Figure 1:**
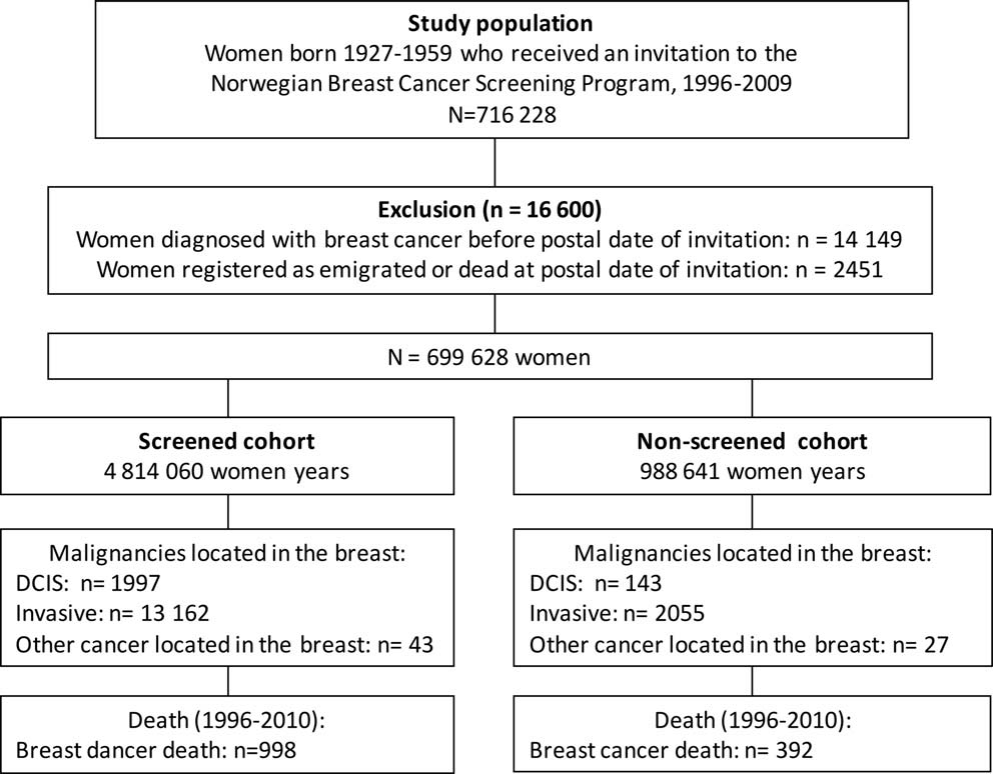
The number of women invited and screened in the Norwegian Breast Cancer Screening Program from 1996 to 2009, exclusions, and final study cohorts, including women-years, the number of breast cancer cases, and the number of breast cancer deaths from 1996 to 2010. DCIS indicates ductal carcinoma in situ.

The NBCSP is administered by the Cancer Registry of Norway and targets women ages 50 to 69 years.^14^ The program started in 4 of 19 counties in 1996 and became nationwide in 2005. The program is run according to European guidelines.^15^ Each woman in the target group receives a personal letter inviting her to undergo 2-view mammography screening every second year, regardless of her cancer history (Fig. 1).

Cancer reporting is mandatory by law in Norway, and the Cancer Registry has registered cancers since 1953.^16^ The database is 99% complete for solid tumors, including breast cancer.^17^ Vital status, date of emigration, and date and cause of death are available from Statistics Norway. This information is regularly linked to the Cancer Registry data by the unique 11-digit personal identification number assigned to all residents of Norway.

We received an anonymized file with individual level dates of invitations and attendance for all women who were invited to the NBCSP and linked this information to the dates of diagnosis of breast cancer, emigration, and death (Fig. 1). The database was created in November 2011. No ethical committee approval was necessary, because we received anonymized data only.

Women were eligible for inclusion if they had been invited to the program during the period from 1996 to 2009. Women who were diagnosed with breast cancer before the postal date of the invitation to the program were excluded (Fig. 1). No organized preliminary clinical breast examination or mammogram screening was performed before the invitation to eliminate prevalence cancers from the population before attendance in the NBCSP. Women were followed until emigration, death, or the end of follow-up (December 31, 2010), whichever date came first. Women who were invited to the NBCSP were defined as screened or nonscreened based on the date of their first attendance in the program. A woman contributed with women-years in the nonscreened cohort from the postal date of her first invitation to the date of her first screening attendance (or, for women who were diagnosed with cancer, until the end of follow-up), whereas a woman contributed with women-years in the screened cohort from the date of her first screening attendance to the end of follow-up. Only women who were free from breast cancer could change their status from nonscreened to screened.

We used a never/ever screened approach in which the women were considered ever screened after their first screening attendance. The International Classification of Diseases, Ninth Revision (ICD-9)/ICD-10 definition of breast cancer was used as the underlying cause of death.^18^ In Norway, cause of death is reported in 3 levels: immediate, intermediate, and the underlying cause of death is reported.^19^ To correct for errors and irrational conclusions drawn by the physician, rules from the World Health Organization are used to ensure correct classification on the basis of the death certificate. Until 2005, the underlying cause of death was determined manually; however, since 2005, the underlying cause of death has been determined electronically. The electronic version requires that the cause of death must fit into ICD-10 codes.^19^

### Statistics

We used Poisson regression to estimate the mortality rate ratio (MRR) of breast cancer death among women who attended in the program compared with those who did not attend. We adjusted for calendar period (continuous), attained age (continuous), and years since inclusion in the screened and nonscreened cohort (in 3-year categories). Using a categorical variable for calendar period and including county of residence in the model altered the MRR to ≤0.5%. Thus, we retained the continuous variable for calendar period and did not include county in the model.

Screened women are expected to have lower breast cancer mortality than those who do not attend screening simply because those who let themselves be screened are more conscious about their health or lifestyle.^20^,^21^ We adjusted for this self-selection bias using the following formula provided by Duffy et al^20^: Ψ′ = Ψ (*pD*/[1 − (1 − *p*)*D*]), where Ψ′ is the parameter of interest (ie, the estimated MRR of breast cancer death adjusted for selection bias), *p* is the proportion of women complying with the invitation to screening; Ψ is the estimated MRR of breast cancer death for compliers versus noncompliers; and *D* is the MRR of breast cancer death for noncompliers compared with uninvited women.^20^

In our study, *p* was used to indicate the percentage of ever-screened women from 1996 to 2009, and Ψ was the adjusted MRR for screened compared with nonscreened women, as indicated in Table1. Because this study only included invited women, the MRR of breast cancer death for noncompliers compared with uninvited women (*D*) was not available. Therefore, we used the average estimate of 1.36 with a 95% confidence interval (CI) of 1.11 to 1.67, from the article by Duffy and colleagues.^20^ The Stata statistical software package (version 12.1; Stata Corporation, Station, Tex) was used for the analyses. We used 2-sided *P* values and a statistical significance level of .05.

**Table 1:** Number of Invasive Breast Cancer Cases, Number of Breast Cancer Deaths, Follow-Up in Women-Years, Crude Breast Cancer Mortality Rates, and Adjusted Breast Cancer Mortality Rate Ratios Associated With Screening in the Norwegian Breast Cancer Screening Program, 1996–2010

					MRR (95% CI)		
Cohort	No. of Invasive Breast Cancer Casess	No. of Breast Cancer Deaths	Follow-Up, Women-Years	Crude Breast Cancer Mortality Rate (95% CI), ×10^−5^	Crude Breast Cancer Mortality	Adjusted Breast Cancer Mortality^a^	Adjusted for Self-Selection Bias: Breast Cancer Mortality^b^
Nonscreened	2055	392	988,641	39.7 (35.8–43.8)	1.00	1.00	1.00
Screened	13,162	998	4,814,060	20.7 (19.5–22.1)	0.52 (0.47–0.59)	0.39 (0.35–0.44)	0.57 (0.51–0.64)

Abbreviations: CI, confidence interval; MRR, mortality rate ratio.

a Poisson regression was adjusted for calendar period (continuous), attained age (continuous), and time since inclusion in the group (categorical; 3-year groups).

b Poisson regression was adjusted for self-selection bias (see Materials and Methods) and for calendar period (continuous), attained age (continuous), and time since inclusion in the group (categorical; 3-year groups).

## RESULTS

From 1996 to 2009, 699,628 women with no history of breast cancer received at least 1 invitation to the screening program in Norway, and 588,982 attended once or more, resulting in a compliance rate of 84%. The average time in the screened and nonscreened cohorts for the women who were diagnosed with cancer was 9.7 years (median, 9.3 years) and 8.6 years (median, 8.2 years), respectively. The average time from diagnosis of breast cancer to the end of follow-up was 5.7 years (median, 5.1 years) for screened women and 5.1 years (median, 4.5 years) for nonscreened women.

**Figure 2:**
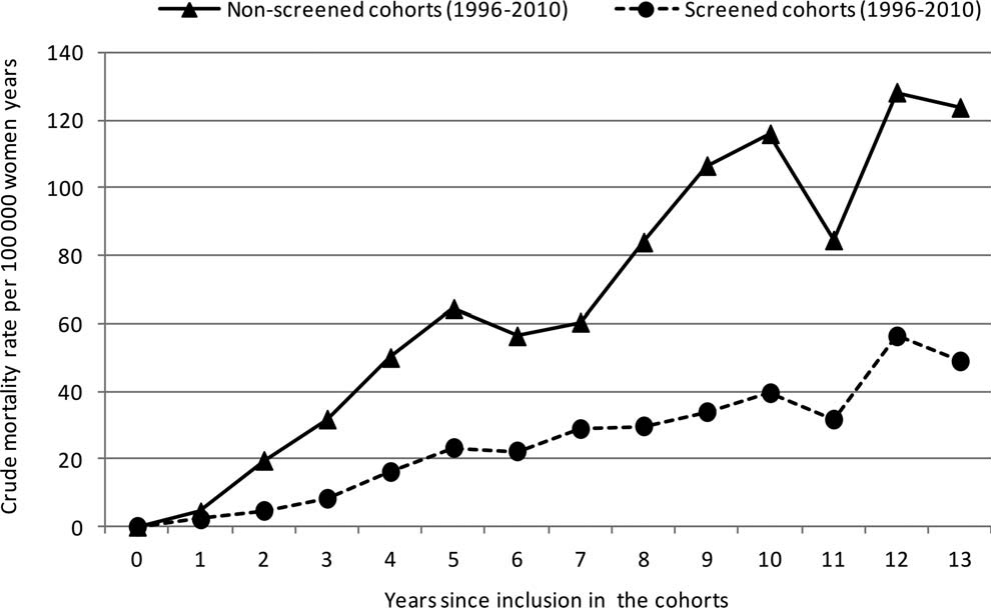
Crude breast cancer mortality rates are illustrated for the screened and nonscreened cohorts of women who were invited to the Norwegian Breast Cancer Screening Program according to the time since inclusion in the cohorts from 1996 to 2010.

**Figure 3:**
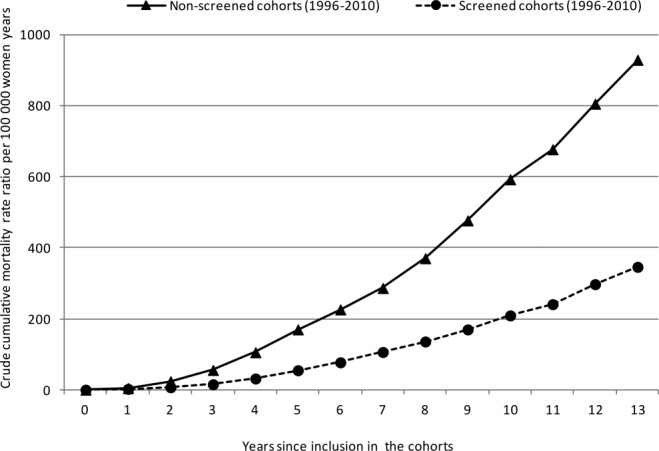
Crude cumulative breast cancer mortality rates are illustrated for the screened and nonscreened cohorts of women who were invited to the Norwegian Breast Cancer Screening Program according to the time since inclusion in the cohorts from 1996 to 2010.

Crude breast cancer mortality rates were 20.7 and 39.7 per 100 000 women-years in the screened and nonscreened cohorts, respectively, which resulted in a crude MRR of 0.52 (95% CI, 0.47-0.59) (Table1). A statistically significant increase in MRR was observed according to attained age and years since inclusion in the cohorts, but not according to calendar period (results not shown). The MRR was 0.39 (95% CI, 0.35-0.44) when adjusted for calendar period, attained age, and years since inclusion in the cohorts (Table1). After adjustment for self-selection bias, we obtained an MRR of 0.57 (95% CI, 0.51-0.64). An MRR of 0.75 (95% CI, 0.67-0.84) was achieved using the more conservative upper 95% CI value for *D* of 1.67 from Duffy et al,^20^ whereas using the lower 95% CI value for *D* of 1.11 resulted in an MRR of 0.44 (95% CI, 0.39-0.49). The difference in crude mortality rates between the 2 cohorts tended to increase with time since inclusion in the cohorts and reached a statistically significant difference after 2 years (Table2; Figs. 2,3).

**Table 2:** Number of Women-Years, Number of Breast Cancer Deaths, Crude Breast Cancer Mortality Rates, and Crude Cumulative Breast Cancer Mortality Rate per 100,000 Women-Years by Time Since Inclusion in the Screened and Nonscreened Cohorts of the Norwegian Breast Cancer Screening Program, 1996–2010

	Screened Cohort				Nonscreened Cohort			
Years Since Inclusion in the Cohorts	Women-Years	No. of Breast Cancer Deaths	Crude Breast Cancer Mortality Rate per 100,000 Women-Years (95% CI)	Crude Cumulative Breast Cancer Mortality Rate per 100,000 Women-Years (95% CI)	Women-Years	No. of Breast Cancer Deaths	Crude Breast Cancer Mortality Rate per 100,000 Women-Years (95% CI)	Crude Cumulative Breast Cancer Mortality Rate per 100,000 Women-Years (95% CI)
1	588,149	13	2.2 (1.2–3.8)	2.2 (1.0–3.4)	348,270	15	4.3 (2.4–7.1)	4.3 (2.1–6.5)
2	573,665	27	4.7 (3.1–6.9)	6.9 (3.9–9.9)	102,719	20	19.5 (11.9–30.1)	23.8 (13.1–34.5)
3	543,231	45	8.3 (6.0–11.1)	15.2 (9.8–20.6)	91,511	29	31.7 (21.2–45.5)	55.5 (33.2–77.7)
4	512,175	83	16.2 (16.2–16.2)	31.4 (22.5–40.3)	82,104	41	49.9 (35.8–67.7)	105.4 (67.9–142.9)
5	480,485	112	23.3 (19.2–28.1)	54.7 (41.5–67.9)	74,635	48	64.3 (47.2–85.7)	169.7 (114.0–225.5)
6	443,308	99	22.4 (18.2–27.2)	77.0 (59.5–94.7)	67,546	38	56.3 (39.8–77.2)	226.0 (152.3–299.6)
7	393,838	114	28.9 (23.9–34.8)	106.0 (83.1–128.9)	58,064	35	60.3 (42.0–83.8)	286.3 (192.7–379.8)
8	336,342	100	29.7 (24.2–36.2)	135.7 (107.0–164.5)	46,491	39	83.9 (59.7–114.7)	370.1 (250.2–490.0)
9	259,792	88	33.9 (27.2–41.7)	169.6 (133.8–205.4)	33,798	36	106.5 (74.6–147.5)	476.7 (321.9–631.4)
10	194,721	77	39.5 (31.2–49.4)	209.1 (164.5–253.8)	24,162	28	115.9 (77.0–167.5)	592.5 (394.9–790.2)
11	154,740	49	31.7 (23.4–41.9)	240.8 (187.3–294.3)	18,927	16	84.5 (48.3–137.3)	677.1 (438.0–916.1)
12	129,748	73	56.3 (44.1–70.4)	297.1 (230.7–363.5)	15,598	20	128.2 (78.3–198.0)	805.3 (510.0–1100.6)
13	114,605	56	48.9 (36.9–64.5)	345.9 (266.7–425.2)	13,747	17	123.7 (72.0–198.0)	929.0 (574.9–1280.3)
14	70,636	41	58.0 (41.7–78.7)	404.0 (307.0–501.0)	8803	7	79.5 (32.0–163.8)	1008.5 (595.5–1421.2)
15	18,626	21	112.7 (69.8–172.3)	516.7 (371.5–661.9)	2265	3	132.5 (27.3–387.1)	1140.9 (578.1–1703.7)

Abbreviations: CI, confidence interval.

## DISCUSSION

This is the first mortality analysis of the national screening program in Norway using individual level screening data. Fifteen years after the start of the program, the screened cohort had a 43% lower breast cancer mortality rate compared with the nonscreened cohort. These results are concordant with studies in other service screening programs and with the analyses from the Euroscreen review, which reported reductions from 38% to 48% in breast cancer mortality among screened women compared with nonscreened women.^4^,^5^

We observed a lower mortality rate in the screened cohort versus the nonscreened cohort as early as the second year after inclusion in the cohorts (Table2). Data from randomized controlled trials have indicated that cumulative breast cancer mortality rates start diverging 4 years after randomization,^22^ but it has been argued (most recently by the independent UK panel) that at least 10 years of follow-up are needed to observe maximum effect.^3^ Because our findings were based on a service screening program with no randomization, self-selection bias may explain the division of the curves as early as 2 years after randomization. Such self-selection bias is caused by the underlying differences between attendees and nonattendees to the screening program that would also be present in the absence of screening. Women who attend a screening program are expected to have a lower risk of breast cancer death compared with nonattendees because of personal characteristics, such as health awareness, family history of breast cancer, and use of mammography before the screening program.^20^,^21^

To adjust for self-selection bias, we used the model described by Duffy et al.^20^ Using the value *D* (the MRR of breast cancer death in noncompliers [nonscreened] compared with the noninvited) from that publication may be considered a limitation of our study, because that value was based on randomized controlled trials and was not from the NBCSP.^23^ Our estimate of the screening effect may be overestimated if *D* is larger in the NBCSP than in the randomized controlled trials, and vice versa. However, other European service screening programs have reported values for *D* of 1.11 and 1.17,^24^,^25^ which are similar to the lower level of the 95% CI for *D* used in our study. However, even if we used a value of 1.67 for *D*, the upper value of the 95% CI for *D* in our adjustment for self-selection bias, which we believe is unreasonably high for Norway; the mortality reduction is still 25% in favor of screening. We did not estimate *D* in our own data because we did not have access to individual level data on women who were not invited to the program.

The objective of our study was to estimate the mortality reduction in women who were screened in the NBCSP, and not the effect of mammography in Norwegian women in general. Use of mammography outside the program, both among nonscreened women and in between 2 screening rounds among screened women, could have reduced the estimated mortality reduction in our study. In Norway, 38% of the prevalently screened women during the period from 1996 to 2006 reportedly had received a mammogram within the last 3 years, whereas 64% reported ever use of mammography before attending the program (results not shown). Furthermore, a recently published study from 1 of the 19 counties in Norway indicated that 32% of the cancers detected in invited, nonattending women were asymptomatic.^26^ The mortality reductions would likely have been even greater in our study if data on such private screening had been available, so that a comparison could have been made between women who participated in the NBCSP and women who were never screened, either privately or as part of the NBCSP.

Breast cancer treatment improved after the start-up of the NBCSP because of the contemporary establishment of breast clinics.^12^ However, this should not to have biased the estimates in our study, because only women who were invited to the program were included. Furthermore, socialized medicine and nationwide guidelines for breast cancer treatment^27^ increase the probability, but do not guarantee, that women throughout the country receive similar treatment. Although we cannot completely exclude the possibility that the screened women received better treatment than nonscreened women, we believe this is unlikely. It is also unlikely that such a bias would have caused the divergence of MRRs over time.

It can be argued that, to assess the effect of a screening program on a population level, breast cancer mortality in invited women should be compared with that of noninvited women. Two previous studies estimated reductions in breast cancer mortality of 10% and 11% for NBCSP-invited women versus noninvited women.^9^,^13^ Those studies were limited by a lack of individual screening data and short follow-up and, thus, probably underestimated the true effect of screening on mortality.

To correctly evaluate the effectiveness of the NBCSP, individual level data on never-invited women would be needed. Such information is currently not available. Because we had access to individual level data on women who were invited to the NBCSP, we focused on mortality in ever-screened women versus never-screened women. However, the mortality reduction on a population level corresponds to the efficacy (mortality reduction in screened vs nonscreened women) multiplied by the compliance rate.^28^,^29^ By using our estimate for mortality reduction (43%) and a compliance rate of 84%, our estimate of the mortality reduction on the population level is 36% (43% × 84%) in favor of the invited women.

The average follow-up for the screened cohort was substantially longer than that for the nonscreened cohort (Table1). This was because >70% of the women who attended the screening program did so within 2 weeks of the first invitation.^30^ However, our model included adjustments for time since inclusion in the cohorts.

Our observational cohort study has several strengths. We used individual level data on invitation and attendance in the program as well as on the outcome of the screening examination. The Cancer Registry of Norway is essentially complete in terms of breast cancer reporting.^17^ In conclusion, the results from this first mortality analysis based on individual level data from the NBCSP, in which women ages 50 to 69 years were invited to biennial screening, indicate a substantial reduction in mortality from breast cancer in screened women compared with nonscreened women.

## FUNDING SUPPORT

No specific funding was disclosed.

## CONFLICT OF INTEREST DISCLOSURES

All authors are employed at the Cancer Registry of Norway, which is administering the Norwegian Breast Cancer Screening Program.
